# Supplementary material for: Protective Effect of Ferulic Acid against Hydrogen Peroxide Induced Apoptosis in PC12 Cells
Source: Molecules. 2020 Dec 28;26(1):90. doi: 10.3390/molecules26010090 (PMC7795901; doi:10.3390/molecules26010090)
Supplement: Supplementary file 1 [file molecules-26-00090-s001.pdf]

Protective Effect of Ferulic Acid against Hydrogen Peroxide-induced Apoptosis in PC12 cells

Authors: Hironao Nakayama [1,2,†,*], Masako Nakahara [1,†], Erina Matsugi [1], Midori Soda [2], Tomoka Hattori [2], Koki Hara [2], Ayuki Usami [2], Chiaki Kusumoto [1], Shigeki Higashiyama [3], and Kiyoyuki Kitaichi [2,*]

Affiliations:
[1]Department of Medical Science and Technology, Hiroshima International University, Higashi-hiroshima, Hiroshima 739-2695, Japan
[2]Laboratory of Pharmaceutics, Gifu Pharmaceutical University, Gifu, Gifu 501-1196, Japan
[3]Division of Cell Growth and Tumor Regulation, Proteo-Science Center, Ehime University, Toon, Shitsukawa, Ehime 791-0295, Japan

[*]Hironao Nakayama and Kiyoyuki Kitaichi served as co-senior authors.
[†]These authors contributed equally to this work, authorship order was determined randomly.

[*]Correspondence: Hironao Nakayama, PhD., Department of Medical Science and Technology, Hiroshima International University, 555-36 Kurose-gakuendai, Higashi-hiroshima, Hiroshima 739-2695, Japan.
E-mail: hironao@hirokoku-u.ac.jp; Tel.: +81 (823) 70-4640

SUPPLEMENTARY FIGURE 1.

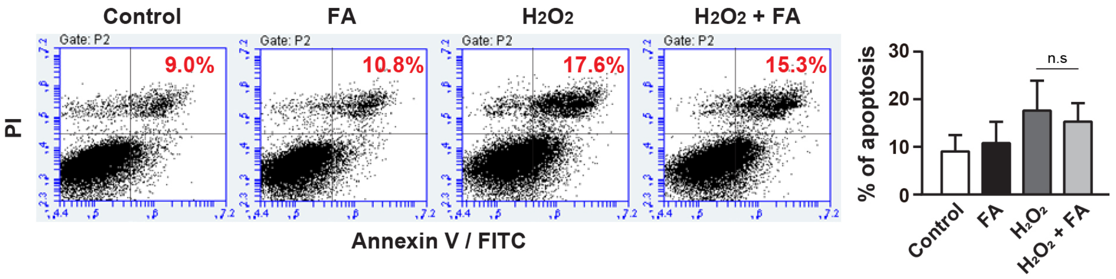

Figure S1. Effect of FA against oxidative stress in PC12 cells. PC12 cells were pretreated with FA (40 µM) overnight and subsequently cultured cells in the absence or presence of H_2O_2 (1 mM) for another 30 min. Cells were stained with Annexin V-FITC/PI to detect apoptotic cells (upper right quadrant) by flow cytometry. Data represent the mean ± SD.

SUPPLEMENTARY TABLE 1.: List of apoptotic proteins modulated by FA.

Spot	Target	ratio (FA+H_2O_2 / FA)
A1,A2	Reference Spot	95.1
A23,A24	Reference Spot	104.5
B1,B2	Bad	53.0
B3,B4	Bax	84.8
B5,B6	Bcl-2	86.9
B7,B8	Bcl-x	70.7
B9,B10	Pro-Caspase-3	89.2
B11,B12	Cleaved Caspase-3	93.3
B13,B14	Catalase	95.7
B15,B16	cIAP-1	99.2
B17,B18	cIAP-2	94.5
B19,B20	Claspin	92.2
B21,B22	Clusterin	90.9
B23,B24	Cytochrome c	91.7
C1,C2	Trail R1/DR4	95.3
C3,C4	Trail R2/DR5	93.2
C5,C6	FADD	93.6
C7,C8	Fas/TNFSF6	96.4
C9,C10	HIF-1alpha	89.6
C11,C12	HO-1/HMOX1/ HSP32	96.3
C13,C14	HO-2/HMOX2	99.3
C15,C16	HSP27	97.8
C17,C18	HSP60	69.9
C19,C20	HSP70	90.9
C21,C22	HTRA2/Omi	96.3
C23,C24	Livin	95.4
D1,D2	PON2	94.3
D3,D4	p21/CIP1/CDNK1A	91.0
D5,D6	p27/Kip1	96.0
D7,D8	phospho-p53 (S15)	90.4
D9,D10	phospho-p53 (S46)	95.3
D11,D12	phospho-p53 (S392)	95.7
D13,D14	phospho-Rad17 (S635)	93.3
D15,D16	SMAC/Diablo	62.1
D17,D18	Survivin	103.2
D19,D20	TNF RI/TNFRSF1A	97.3
D21,D22	XIAP	78.8
D23,D24	PBS (Negative Control)	N/D
E1,E2	Reference Spot	95.8

SUPPLEMENTARY TABLE 2.: List of phospho-kinase proteins modulated by FA.

Membrane	Spot	Target	ratio (FA+H2O2 / FA)
A	A1,A2	Reference Spot	92.3
A	A23,A24	p38 alpha (T180/Y182)	86.4
A	A5,A6	ERK1/2 (T202/Y204, T185/Y187)	70.6
A	A7,A8	JNK 1/2/3 (T183/Y185, T221/Y223)	91.4
A	A9,A10	GSK-3 alpha/beta (S21/S9)	88.9
B	A13,A14	p53 (S392)	91.9
B	A17,A18	Reference Spot	92.7
A	B3,B4	EGFR (Y1086)	79.9
A	B5,B6	MSK1/2 (S376/S360)	80.3
A	B7,B8	AMPK alpha1 (T183)	99.7
A	B9,B10	Akt (S473)	95.4
B	B11,B12	Akt (T308)	82.4
B	B13,B14	p53 (S46)	76.5
A	C1,C2	TOR (S2448)	78.9
A	C3,C4	CREB (S133)	87.2
A	C5,C6	HSP27 (S78/S82)	90.2
A	C7,C8	AMPK alpha2 (T172)	100.1
A	C9,C10	beta-Catenin	95.1
B	C11,C12	p70 S6 Kinase (T389)	82.4
B	C13,C14	p53 (S15)	76.5
B	C15,C16	c-Jun (S63)	92.9
A	D1,D2	Src (Y419)	73.6
A	D3,D4	Lyn (Y397)	89.8
A	D5,D6	Lck (Y394)	88.5
A	D7,D8	STAT2 (Y689)	97.9
A	D9,D10	STAT5a (Y694)	105.1
B	D11,D12	p70 S6 Kinase (T421/S424)	83.2
B	D13,D14	RSK1/2/3 (S380/S386/S377)	93.1
B	D15,D16	eNOS (S1177)	90.2
A	E1,E2	Fyn (Y420)	81.9
A	E3,E4	Yes (Y426)	100.6
A	E5,E6	Fgr (Y412)	85.7
A	E7,E8	STAT6 (Y641)	104.7
A	E9,E10	STAT5b (Y699)	116.7
B	E11,E12	STAT3 (Y705)	89.8
B	E13,E14	p27 (T198)	86.4
B	E15,E16	PLC-g1 (Y783)	99.8
A	F1,F2	Hck (Y411)	77.8
A	F3,F4	Chk-2 (T68)	105.5
A	F5,F6	FAK (Y397)	111.4
A	F7,F8	PDGF Rb (Y751)	97.5
A	F9,F10	STAT5a/b (Y694/Y699)	107.8
B	F11,F12	STAT3 (S727)	85.4
B	F13,F14	WNK1 (T60)	84.1
B	F15,F16	Pyk2 (Y402)	103.7
A	G1,G2	Reference	97.5
A	G3,G4	PRAS40 (T246)	116.9
A	G9,G10	PBS (Negative Control)	N/D
B	G11,G12	HSP60	98.9
B	G17,G18	PBS (Negative Control)	N/D
